# Oxygen saturation and blood volume analysis by photoacoustic imaging to identify pre and post-PDT vascular changes

**DOI:** 10.1016/j.sjbs.2022.103304

**Published:** 2022-04-22

**Authors:** M. Atif, Atif Hanif, M.S. AlSalhi, S. Devanesan, Haya Abdulaziz Altamimi

**Affiliations:** a Department of Physics and Astronomy, College of Science, King Saud University, Riyadh 11451, Saudi Arabia; b Botany and Microbiology Department, College of Science, King Saud University, Riyadh 11451, Saudi Arabia

**Keywords:** Photoacoustic imaging, Bioluminescence, Blood volume, Aminolevulinic acid, Benzoporphyrin derivative

## Abstract

In this study, the blood volume and oxygen saturation of tumors were measured after photoacoustic imaging (PAI) under conditions of pre-photodynamic therapy (PDT), post-PDT, and 4 hrs, and 24 hrs post-PDT. PDTs with aminolevulinic acid (ALA) and low and high doses of benzoporphyrin derivative (BPD) were conducted to observe oxygen saturation changes, and the rapid oxygen consumption in the blood detected due to the action of BPD at the vascular level resulted in the recovery of PDT completion. Likewise, blood volume changes followed by ALA-PDT and BPD-PDT at low and high doses depicted a fast expansion of the blood volume after treatment. The tumor subjected to a high dose of ALA-PDT showed a partial alteration of Hb-pO_2_ in the first 24 hrs, as did the tumors treated with two ALA- and BPD-mediated PDTs. The Hb-pO_2_ started reducing immediately post-PDT and was less than 30% after 4 hrs until 24 hrs post-PDT. Reduced vascular demand was possibly due to tumor necrosis, as shown by the permanent damage in the cancer cells' bioluminescence signal. The ALA-mediated PDT-subjected tumor showed a 50% drop in BV at 24 hrs post-PDT, which is suggestive of vascular pruning. The studied data of blood volume against BLI showed the blood volume and oxygenation variations validating the cells' metabolic activity, including cell death.

## 1 Introduction

PDT, or the use of light-activated drugs (photosensitizers), is delivered in a single minimally invasive treatment and is approved for some cancers. It has no systemic toxicity and hence also had only minimal, short-term side effects and minimized overall treatment duration and recovery time. However, PDT outcomes vary but are excellent for most skin cancers. Using the current state-of-the-art treatment protocols, the response of other cancers, such as those in the brain or lungs, is variable, so a high fraction of long-term survivors has been demonstrated. Patient selection for PDT is heuristic and not based on objective measures of their suitability, particularly if a vascular or cellular acting photosensitizer is preferable. The state of the treatment planning considers only light distribution but rarely photosensitizer accumulation. Photoacoustic imaging is an established technique that allows the imaging of large tissue volumes in the presence of strong absorbers such as blood (Agostinis et al., 2011, Brooksby et al., 2006, Attia et al., 2019, Guo et al., 2020, Laufer et al., 2012, Leng, 2022, Lin et al., 2014, Liu et al., 2013, Cao et al., 2017, Maneas et al., 2020, Manwar et al., 2020, Qiu et al., 2021, Zhou and Jokerst, 2020).

Personalized cancer therapy will enable physicians to match a particular treatment to each of their patients and their particular tumor based on location and genetic expression. Starting a particular course of treatment typically takes considerable time until the cancer’s response is established, delaying the use of alternative therapies. While PDT comprises a single therapy session and should thus present a preferable treatment option, its use by oncologists is limited, as they lack an objective patient selection criterion and need strong outcome prediction measures soon after therapy (Mallidi et al., 2015, Maslov et al., 2008, Sethuraman et al., 2008, Standish et al., 2007, Wang and Hu, 2012, Yang et al., 2009, Yu et al., 2005, Atif et al., 2005, Atif, 2012, Atif et al., 2016).

A mature imaging technique to be applied prior to and after PDT will address both of these shortcomings to ultimately promote the use of PDT as a minimally invasive therapy for those patients who are highly likely to benefit from it. Enabling the use of minimally invasive single-session therapies provides cancer patients with new options for treatment and ultimately significantly increases their quality of life (Kim et al., 2010a, Kim et al., 2010b, Valdés et al., 2011, Valdés et al., 2012, Kim et al., 2010b).

The current study aims to exploit photoacoustic imaging, an optical/ultrasonic technique, to determine a) a patient’s suitability for PDT, b) the local photosensitizer accumulation, and c) the tumor response within days of treatment. We will evaluate the feasibility of noninvasive photoacoustic imaging (a combined optical/ultrasound imaging technique) to determine a tumor's ability to accumulate an adequate amount of photosensitizer and provide oxygen prior to and during treatment. This will be combined with existing simulations of light distribution in the target tissue to maximize tumor response. We studied the blood volume and oxygen saturation using PAI under conditions of pre-PDT, post-PDT, and 4 hrs and 24 hrs post-PDT. The effects of aminolevulinic acid (ALA)-PDT and low and high doses of benzoporphyrin derivative (BPD) were studied against rapid oxygen consumption in the blood and oxygen saturation changes. We observed local changes in the tumor vasculature and compared them to tumor survival post-PDT in vivo models.

## 2 Materials and methods

The human prostate cancer cell line PC3 (Lin et al., 2014, Liu et al., 2013) was grown as subcutaneous (SC) xenografts on both hind limbs of nude mice and induced with 5 million cells in 100 µL of phosphate-buffered saline (PBS). When tumors reached 5–8 mm in diameter, the mice were imaged with PAI followed by injection with one PS, followed by PDT with a 7-day follow-up imaging. ALA (60 mg/kg) and low (1 mg/kg) and high (3 mg/kg) concentrations of BPD were utilized as photosensitizers. Light activation started and 4 hrs or 15 min post-photosensitizer administration for ALA and BPD, respectively. Tumors were imaged at multiple λ by the PAI system, initially at 780 and 830 nm as the minimum wavelengths, in order to determine the deoxyhemoglobin signals over the oxyhemoglobin signals to acquire the blood volume and oxygen saturation. The animals were euthanized, and the tumors were immediately removed and placed onto ice for imaging. Each tumor was sliced into 4 3-mm-thick layers, aiming for one cut for the 1.5 mm tumor central plane imaged by PAI. SFDI excitation was carried out at 405 nm to 532 nm depending on the photosensitizer, and 3 spatial frequencies from 0.09 to 0.48 mm^−1^ were employed to separate the deeper PS signals from the proximal surface PS signals. The correlation between the PAI imaging of the BV and the SFDI-determined PS concentration was established using orthotopic tumor models. In these studies, animals were imaged with PAI prior to PS administration, and several fresh tumor slices were used for SFDI excitation post-euthanasia at the PS-specific delay times.

**Fig. 1:**
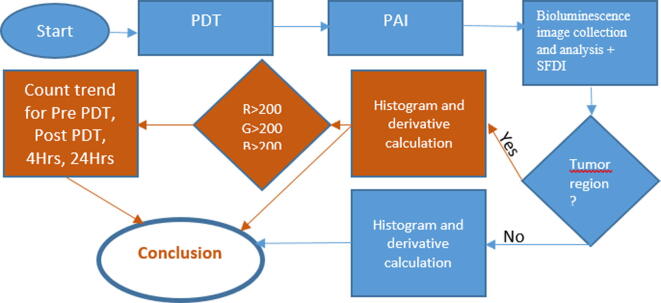
Schematic detailed diagram of the experimental plan.

For this study, a few early indicators are proposed as outcomes of the response to photodynamic therapy against tumor cells. After photodynamic therapy, images are taken from different angles using photoacoustic images at different time stamps. These images are later superimposed onto the anatomical images as reported in a previous study (Atif et al., 2021). The response to photodynamic therapy was observed under conditions of pre-PDT, post-PDT, and 4 hrs and 24 hrs post-PDT. These spatial domain figures were analyzed using thresholding and by the counting of the cells having values greater than the threshold. A tumor area is selected for the analysis from the whole image. Similarly, an area outside the tumor region was also selected for comparison with the tumor region. The image is further decomposed to its constituent channels by color scales, i.e., red, green and blue channels. The block diagram shown in Fig. 1 describes the whole process. The spatial domain image is then converted to a grayscale image. Histograms and derivatives of histograms for tumor region images were calculated for each case of pre-PDT, post-PDT, and 4 hrs and 24 hrs post-PDT. Similarly, histograms and derivatives of histograms for normal region images are calculated for each case of pre-PDT, post-PDT, and 4 hrs and 24 hrs post-PDT. The spatial figures are also converted into frequency domains to show the frequency contents present in the tumors and normal tissues as observed by PAI.

## 3 Results

Fig. 1 shows the block diagram of the research that yielded the experimental results used to reach a valid conclusion.

Each channel of red, blue and green is observed over time under the conditions of pre-PDT, post-PDT, and 4 hrs and 24 hrs post-PDT. The color information is collected from the image presented in Fig. 1 as reported in a previous study (Atif et al., 2021). The image has many colors, but all these colors are combinations of red, blue and green. Blue and green channels are excluded due to the representation of normal tissue area around the tumor. The intensity level threshold is taken as 200, and the red channel pixels are counted with a red channel value over 200 for each pixel. The threshold of the red channel is selected at random. The pixels that are greater than 200 are closer to that color. In summary, the red channel consists of a threshold value above 200. A pixel value greater than 200 means that it is referring to the red color in the image. The red color is important as it shows the maximum intensity. The number of pixels with values larger than 200 for the red channel is listed in Table 1.

**Table 1:** Analysis of the color image of Fig. 1 (Atif et al., 2021) by using the measure of average color intensity by selecting a window at the tumor region and determining the red channel intensities.

Channel	Pre-PDT	Post PDT	4Hrs	24Hrs
Red channel	730	99	52	40

The red channel shows the highest intensity, which has a decreasing trend from pre-PDT to 24 hrs post-PDT. There are two points that are highlighted: First, the change in each derivative of the histogram is greater initially, and with increasing pixel intensity, the change is decreasing, which shows that the image has low frequencies rather than high frequencies. Second, the change from pre-PDT to 24 hrs post-PDT also decreases, which shows that it has the minimum change.

**Fig. 2:**
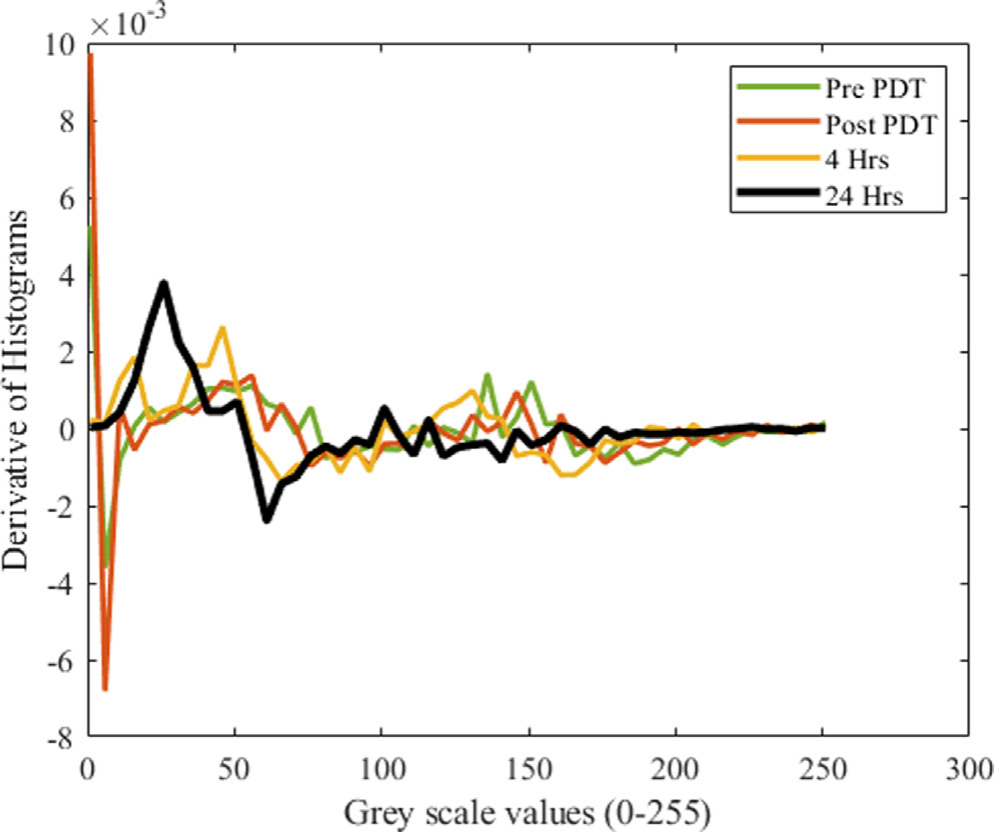
Derivative histograms for the conditions of pre-PDT, post-PDT, and 4 hrs and 24 hrs post-PDT for the tumor region plotted against grayscale images.

Spatial domain images are converted to grayscale images to produce the histograms. The derivatives of the histograms are plotted in Fig. 2.

It is observed that initially, the derivative of the histogram deviates from the zero values for the case of pre-PDT. The deviation from the zero values decreases from lower grayscale values to higher grayscale values. Moreover, the same trend was also found in the other cases of 4 hrs and 24 hrs post-PDT. Correspondingly, the green and blue channels are also found to have similar decreasing trends.

It is also noticed that after 24 hrs post-PDT, the cumulative values of the derivatives of the histograms are close to zero. The trends of the mean variance and sum were calculated, and their trends were decreasing, which might be due to the decrease in the tumor region (Table 2).

**Table 2:** First-order statistics containing the mean, variance and sum values of the derivatives of histogram for the tumor region.

Parameter	Pre-PDT	Post-PDT	4 Hrs	24 Hrs
Mean	0.1255	0.0431	0.0235	0.0078
Variance	1347.16	1265.5	440.225	248.5199
Sum	6.4	2.2	1.2	0.4

Table 2 shows that the mean values of the derivatives of the histogram showed a decreasing trend, showing that the difference between the pixel values decreased from pre-PDT to 24 hrs post-PDT. Similarly, the variance also has a decreasing trend for the derivatives of the histogram. This trend also shows that the pixels in the area result from cells that are becoming normal cells. From the whole images, only tumor regions were selected. The sum of the derivatives of the histograms also shows a decreasing trend.

**Fig. 3:**
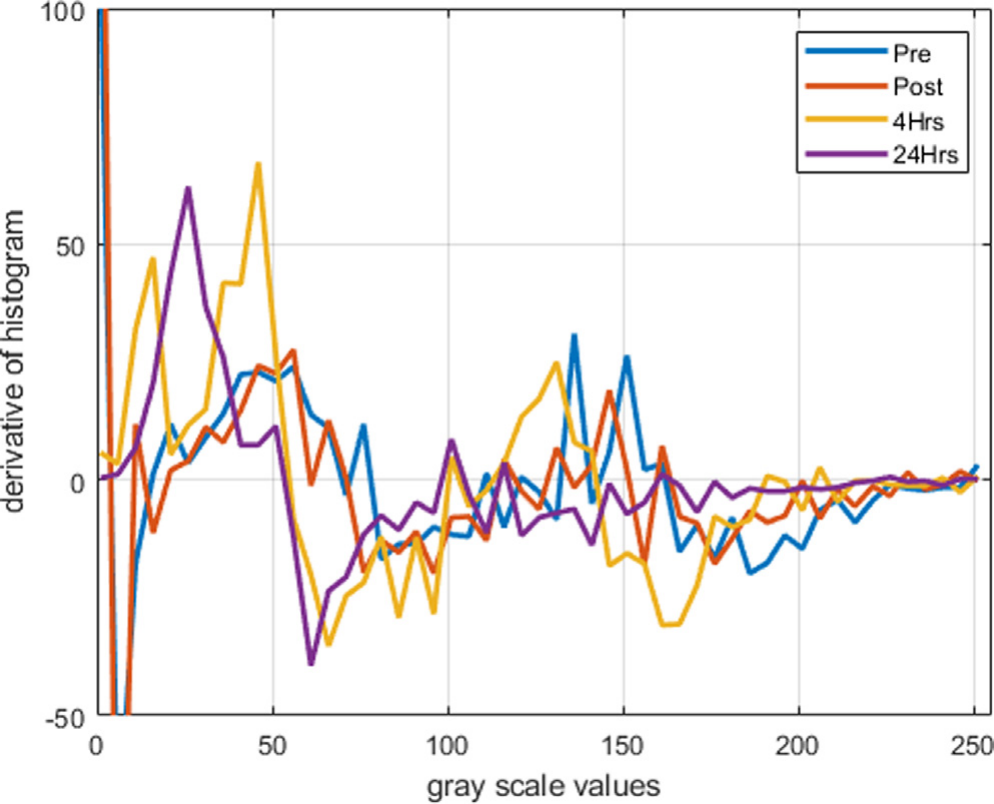
Derivative histograms for pre-PDT, post-PDT, and 4 hrs, and 24 hrs post-PDT for the normal region plotted against grayscale images.

Fig. 3 shows the derivatives of histograms for pre-PDT, post-PDT, and 4 hrs, and 24 hrs post-PDT for the normal region. A similar justification can be developed for these figures as that developed for the case of tumor regression.

Histograms are shown for the frequency domain images in Table 3 for pre-PDT to 24 hrs post-PDT for the tumor region, and the frequency domain images are converted to log scale. Finally, absolute values are shown.

**Table 3:** Sum of the absolute differences in histograms for pre-PDT, post-PDT, and 4 hrs and 24 hrs post-PDT.

Description	Pre PDT	Post PDT	4 Hours	24 Hours
Sum of Absolute Differences in Histogram	0.6533	0.6121	0.4492	0.2197

**Fig. 4:**
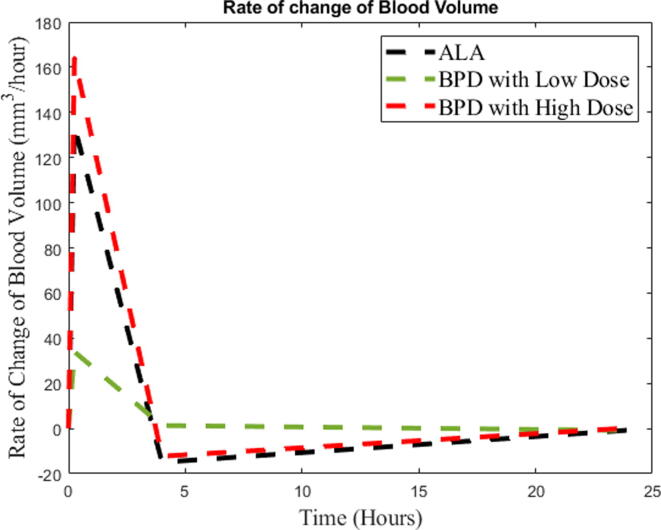
Rate of change in blood volume depending on BPD-PDT at a high dose, BPD-PDT at a low dose and ALA-PDT.

**Fig. 5:**
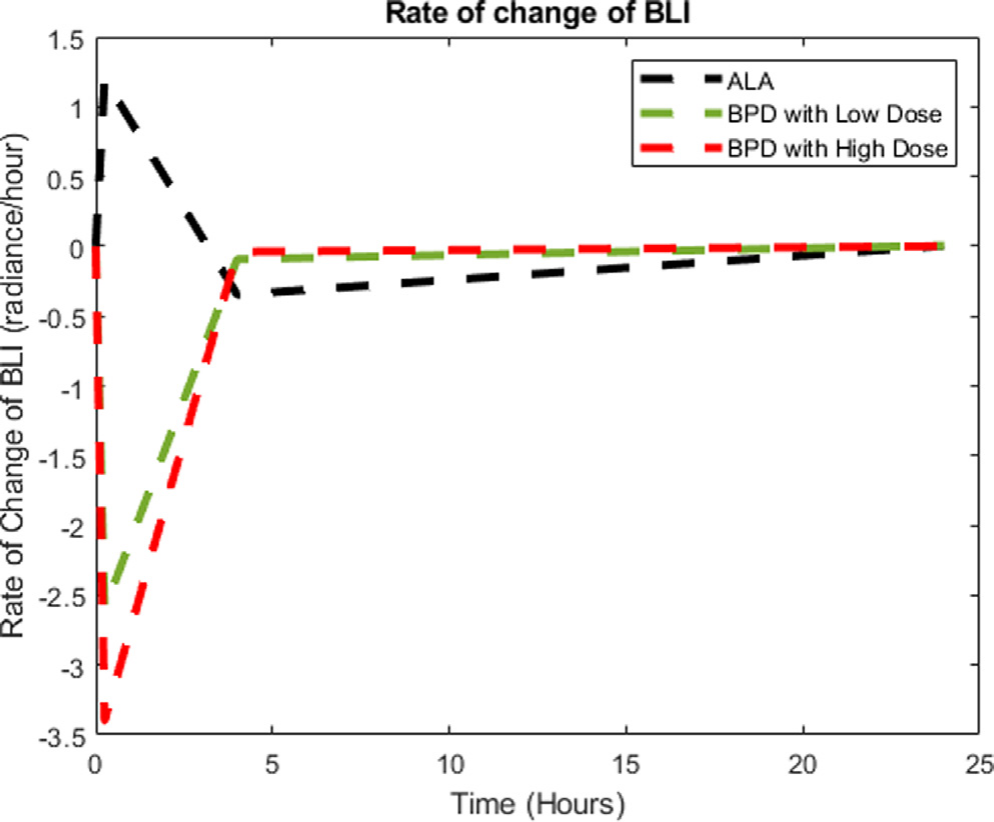
Rate of change of BLI depending on the BPD-PDT at a high dose, BPD-PDT at a low dose and ALA-PDT.

Fig. 4 shows the changes in blood volume for 3 tumors. It can be seen that BPD-PDT at a low dose has the lowest blood volume for all the time values from 0 to 24 hrs, while the blood volume for the BPD-PDT at a high dose has the largest blood volume for all the time values. The data were collected from the blood volume experiment. Different trials are conducted for each of these experiments, such as BPD-PDT at a high dose and PDT at a low dose of BPD and ALA. These experiments were evaluated under conditions of pre-PDT, post-PDT, and 4 hrs and 24 hrs post-PDT. Fig. 5 shows the relative BLI change under conditions of pre-PDT, post-PDT, 4 hrs and 24 hrs post-PDT.

Fig. 5 shows the relative BLI as a function of time. The data are collected from different experiments of relative BLI. Different trials are made for each of these experiments, such as BPD-PDT at a high dose, BPD-PDT at a low dose and ALA-PDT. These experiments were evaluated for pre-PDT, post-PDT, and 4 hrs and 24 hrs post-PDT.

**Fig. 6:**
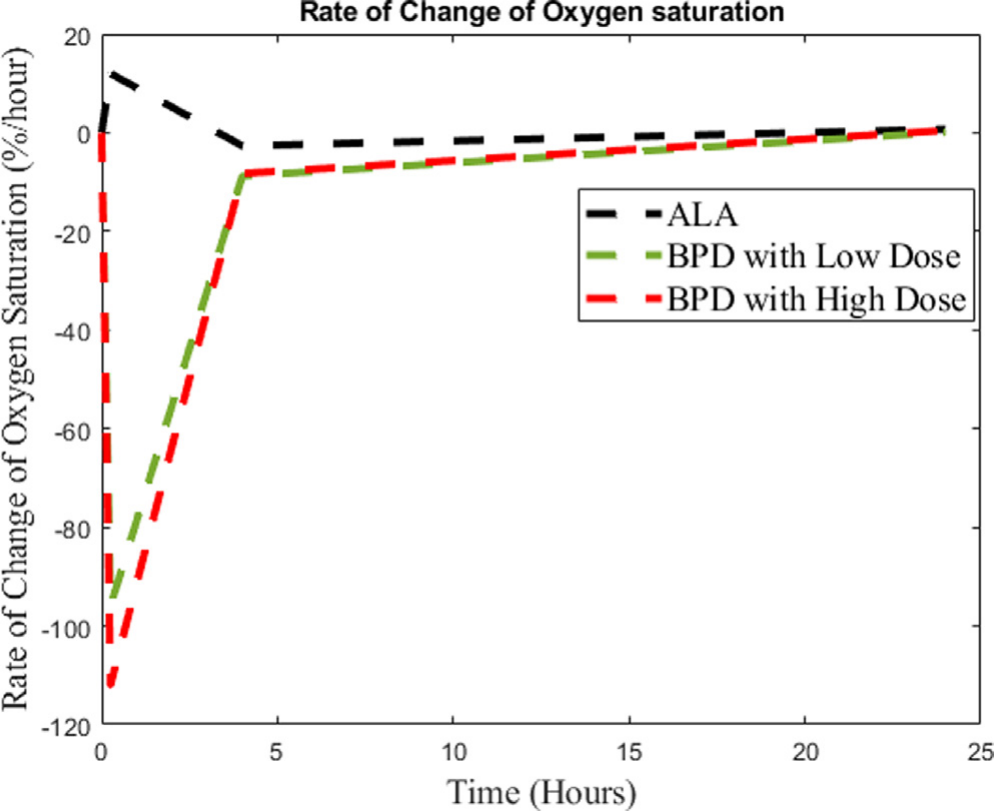
Rate of change of oxygen saturation (%) depending on the BPD-PDT at a high dose, BPD-PDT at a low dose and ALA-PDT.

The experimental results of oxygen saturation (%) depending on BPD-PDT at a high dose, BPD-PDT at a low dose and ALA-PDT are shown in Fig. 6. The experiments were conducted for pre-PDT, post-PDT, and 4 hrs and 24 hrs post-PDT.

## 4 Discussion

As part of a study aimed at enabling personalized photodynamic therapy (PDT), as discussed in previously reported studies (Akens et al., 2007, Fisher and Lilge, 2015, Jalali et al., 2014, Kaspler et al., 2016, Kim et al., 2010a, Konecky et al., 2012, Nakajima et al., 2014, Sunar et al., 2013), the study seeks to exploit the imaging of a tumor’s vasculature to identify cancer patient’s suitability for PDT by providing information on photosensitizer uptake into the tumor, which will assist in PDT planning. Additionally, imaging changes to the tumor’s vasculature post-PDT could predict treatment outcome. By personalizing treatment planning, we will increase the probability of a complete response following PDT through patient selection and PDT dose optimization, and by predicting the response due to vascular changes, we will ensure patients and physicians that their treatment was successful.

In the current study involving photoacoustic imaging (PAI), we evaluated the feasibility of noninvasive photoacoustic imaging to quantify a tumor's perfusion to predict its ability to accumulate an adequate amount of photosensitizer and provide oxygen prior to and during treatment. The spatial photosensitizer and oxygen distribution were combined with existing simulations of light distribution in the target tissue to evaluate patient suitability and to maximize tumor response.

During PAI, we used light of different wavelengths to image the oxygenated and nonoxygenated blood components separately, thus generating high solution maps of the blood volume and blood flow via measurements of the total hemoglobin and oxygen saturation levels.

Various biophotonics groups (Mallidi et al., 2015, Standish et al., 2007, Yu et al., 2005) showed that blood flow (BF) and (BV) change post vascular and cellular PS-mediated treatment. Translation of this information in clinical applications was not practicable with the techniques used, which suffered from partial penetration depth or provided only volume-average knowledge, such as diffuse reflectance tomography (Brooksby et al., 2006). Exploiting the high molar absorption of hemoglobin in spectrally resolved photoacoustic imaging (PAI) (Maslov et al., 2008, Wang and Hu, 2012) and the ability of repeated imaging suggests that response monitoring is viable (Laufer et al., 2012). PAI has a penetration depth equal to that of PDT-activating light and permits hemoglobin species-specific images. PAI utilizes pulsed or frequency domain near-infrared light mainly absorbed by a target component, producing transient local heating (« 1 °C) resulting in the thermoelastic expansion of the target, leading to the emission of acoustic waves detectable by an external ultrasound transducer. PAI provides high resolution (50 µm) 3D absorption maps of the vasculature, as reported in a previous study (Atif et al., 2021).

Spatially resolved BV measurements are obtained by 808 nm light-mediated PAI of the isosbestic point for oxyhemoglobin and deoxyhemoglobin, and Hb-pO_2_ is the ratio of the deoxyhemoglobin signals to the oxyhemoglobin signals collected at 780 nm and 830 nm, respectively.

The technical feasibility of the proposed experiments for post-PDT monitoring was demonstrated in a small number of mice carrying a bioluminescent subcutaneous prostate tumor. In these experiments, tumor Hb-pO_2_ and total Hb images representing BV and active perfusion were collected as a function of time post-PDT to detect surface proximal metabolically active tumor cells. One tumor was treated with high-dose ALA-PDT and showed a limited modification of Hb-pO_2_ in the first 24 hrs (Atif et al., 2021), whereas for two ALA- and BPD-mediated PDT-subjected tumors, Hb-pO_2_ started dropping immediately post-PDT and was less than 30% after 4 hrs until 24 hrs post-PDT, as expected. Post ALA-mediated PDT, vasculature in the surviving tumor fractions remains apparent, whereas BPD-mediated PDT resulted in vascular stasis, with the oxygen demand exceeding the oxygen supply, reducing the Hb-pO_2_ saturation. There was little difference in the Hb-pO_2_ between high and low PS doses, so the latter showed little change in the tumor's BV (Atif et al., 2021). The ALA-mediated PDT-subjected tumor showed a 50% drop in BV at 24 hrs post-PDT, which is suggestive of vascular pruning.

It is noted that the red pixels show the highest intensities, and blue pixels show the lowest intensities of hemoglobin concentration. The decreasing trend in the concentration of hemoglobin may lead to the conclusion that tumor cells behave as normal cells (Fig. 2, Fig. 3).

Similarly, the plot of blood volume against time for the PDT with ALA and low and high doses of BPD is shown in Fig. 4. However, there is an initial rapid expansion of blood volume for the treatments, which is due to the extravasation of red blood cells or hemoglobin from lysed RBCs. This condition was also recovered within 4 hrs post-PDT. Reduced vascular demand was probably due to tumor cell debris, as depicted by the permanent loss of the cancer cells' bioluminescence signal (Fig. 5).

The rate of change of BLI of ALA-PDT and BPD low and high doses with PDT is plotted in Fig. 5. The plots of blood volume (Fig. 4) and BLI (Fig. 5) showed the blood volume and oxygenation changes, demonstrating the cells' metabolic activity, including cell death.

The PDT effects of ALA and low and high doses of BPD on the rate of change in oxygen saturation is shown in Fig. 6. Very rapid oxygen consumption in the blood was observed due to vascular BPD, representing rapid recovery after the completion of PDT.

The current study involved spatially resolved in vivo hypoxia information, and its relationship to cell survival was investigated to enhance the patient's benefit. Photoacoustic imaging was utilized to study the imaging of tumor hypoxia and cell survival for the monitoring of photodynamic therapy. The temporal dynamics of bioluminescence signals and photoacoustic imaging techniques were applied to identify pre- and post-PDT vascular changes. The experimental results included BV and active perfusion recorded as a function of time post-PDT to detect surface proximal metabolically active tumor cells. The ALA-mediated PDT-subjected tumor depicted a 50% drop in BV at 24 hrs post-PDT, indicative of vascular pruning. Image correlation with spatial frequency domain images (SFDI) was used to quantify the PS concentration in the tumor’s central slice (Konecky et al., 2012). The spatial PS concentration was coregistered with the central tumor slice’s PAI-derived BV and oxyHb images, allowing some deformation in the images. Temporal changes in BV and hemoglobin concentrations were evaluated.

In future studies, we will quantify local changes in the tumor vasculature and compare them to tumor survival post-PDT in vivo models.

## 5 Conclusion

The blood volume versus time for the PDT of ALA and low and high doses of BPD resulted in an initial rapid expansion of blood volume for the treatments, which is due to extravasation of red blood cells or hemoglobin from lysed RBCs. The ALA-treated PDT-subjected tumors exhibited a 50% reduction in BV at an interval of 24 hrs post-PDT, which is suggestive of vascular pruning. The data from the current study of blood volume against BLI presented the blood volume and oxygenation changes, validating the cells' metabolic activity, including cell death.

## Declaration of Competing Interest

The authors declare that they have no known competing financial interests or personal relationships that could have appeared to influence the work reported in this paper.
